# Out-of-Pocket Patient Payments for Public Health Care Services in Bulgaria

**DOI:** 10.3389/fpubh.2015.00175

**Published:** 2015-07-08

**Authors:** Elka Atanasova, Milena Pavlova, Wim Groot

**Affiliations:** ^1^Department of Health Economics and Management, Faculty of Public Health, Medical University of Varna, Varna, Bulgaria; ^2^Department of Health Services Research, Faculty of Health, Medicine and Life Sciences, CAPHRI, Maastricht University Medical Center, Maastricht University, Maastricht, Netherlands; ^3^Topinstitute Evidence-Based Education Research (TIER), Maastricht University, Maastricht, Netherlands

**Keywords:** out-of-pocket payments, informal payments, health care services, health care reform, Bulgaria

## Prolonged Transition from a Tax-Based to Insurance-Based Health Care Provision

In Bulgaria, the democratic social and economic reforms began when the new Constitution was adopted in 1991. The communist period prior to this reform was marked by the development of the health care system within an environment of centralized management (1). Health care financing was entirely based on general taxation. Medical services were provided by the state or the municipality. All health care establishments were public institutions as private medical practices were prohibited before 1989. These establishments were allocated an earmarked budget, the size of which was mainly determined on a historical basis. The key factors that determined the allocation of funds were the number of staff and beds. Large staff and a high number of beds were rewarded, and a high level of patient admissions and long hospital stays were common. The reimbursement of health care professionals was in the form of a salary based on employment contracts (2). Health care provision was free of charge at the point of service use. There were only small charges when purchasing prescribed pharmaceuticals and devices outside the health care settings (e.g., at the pharmacy).

With the socio-political changes in 1990s, many elements of the Bulgarian health care model were discredited. A number of problems with the health and demographic status of the population became visible. The failure to cope with the inefficiencies in the health care sector, as well as the poor management and suboptimal use of resources for health care, gradually became more evident (3). The reform of the health care system was put on the government agenda. The main aims of the reforms were the restructuring of the health care financing system, strengthening primary care, and rationalizing the network of outpatient and inpatient facilities. The preparation for the introduction of the health insurance system was accompanied by the adoption of legislative acts that formed the basis for the health care reforms.

The discussion on the need to restructure the centralized tax-based health care system into a social health insurance system started in 1990s and was conducted parallel with the transformation of the country’s economy from a centrally planned economy to a market-based economy (1). However, the health care system was transformed into a social health insurance system only in 2000 after the Bulgarian Parliament adopted the Health Insurance Act of 1998 (2). The reform introduced market principles, decentralization, as well as pluralism in the ownership of the health institutions and the provision of health care services.

## Out-of-Pocket Payments have Become the Main Source of Health Care Financing

The regulatory changes created three major players in the system as follows: (a) citizens as insurance buyers/consumers, (b) outpatient and hospital establishments as providers, and (c) public and private health insurance organizations as third-party payers. The Act of 1998 established the National Health Insurance Fund (NHIF) and defined the relationship between the NHIF, health care consumers and providers. The relationships between the NHIF and health care providers are based on the National Framework Contract. Based on this contract model, providers sign individual contracts with the district branches of the NHIF, namely, the 28 Regional Health Insurance Funds (RHIF). The NHIF interacts with all kinds of providers. The general practitioner (GP) is the central figure in the primary care and acts as a gatekeeper for specialized ambulatory and hospital care. Ambulatory care is provided by specialized outpatient facilities, including individual and group practices, medical and medico-dental centers, diagnostic–consultative centers, and medico-technical laboratories. Hospital care is provided by public and private health establishments divided into multi-profile and specialized hospitals (1).

The reform included the introduction of formal patient charges for public health care services. These charges take the form of co-payments and apply to all levels of medical services, except emergency care. Until 2012, the official fee for each outpatient visit to a GP and medical specialist (after a referral) was equal to 1% of the minimum monthly salary for the country. For hospitalization, fee amounts to 2% of the minimum monthly salary per day for the first 10 days of the hospital stay and it is paid once a year. Since these fees were defined as a percentage of the minimum monthly salary, their amount increased with the rise of the minimum monthly salary in the country. However, in order to reduce the financial burden of the insured people, the Council of Ministers replaced user charges set as a percentage of the minimum monthly salary by fixed co-payments in 2012 (4). The formal co-payments are collected and retained by the providers and their official objective is to improve efficiency in public health care provision (5).

Bulgaria currently has a mixed system of health care financing. Health care is financed from compulsory and voluntary health insurance contributions, taxes, out-of-pocket payments, donations, and external funding. The revenue from general taxation has gradually decreased as the compulsory health insurance revenues increased from 1999 to 2003 (6). However, the diminishing state role in the financing of the health care system has led to a significant increase in out-of-pocket payments, which became the predominant source of revenue in 2000 (1). This trend has continued and at present the main source of revenue for the health care system are out-of-pocket payments. The second largest source of revenue is the social health insurance contributions, which constitute 8% of the individual monthly income. These contributions are shared between the employee and employer at a ratio of 40:60, or are paid individually by the self-employed or unemployed. The third main source of revenue is the funds allocated to the Ministry of Health budget from the central budget. In addition to this, there are informal patient payments, which continue to exist irrespective of the formal charges (7).

## The Burden of Out-of-Pocket Patient Payments is Considerable in Bulgaria

In Bulgaria, the share of out-of-pocket payments has increased substantially, which reflects a common trend in Europe. In 2008, out-of-pocket payments in Bulgaria formed 40% of total expenditure on health, compared to 16% in EU. In 2009, this ratio was 43.4% and in 2010, it was 44.2%. This share is one of the highest in Europe (8, 9). The projection for Bulgaria is that this trend will continue and the share of out-of-pocket payments on health care is expected to become as high as 48–49% of total health care expenditures in the coming years (9). Thus, out-of-pocket payments constitute a major source of health financing in Bulgaria and the role of patient payments will become even more important in the future.

A recent study among a nationally representative sample of the population in 2010 and in 2011 (10) has explored whether user fees, combined with informal payments are affordable for the population. Two indicators of inability to pay, namely, the need to borrow money to pay for health care and the need to forego health care services due to high payment requirements, are analyzed. The results show that in 2010 and 2011, 60% of users paid out-of-pocket payments for both physician services and hospitalizations. Of those who paid, about 6% borrowed money to pay for physician services in both years and more than 10% of users borrowed money to pay for hospitalization. In addition to this, 32% of the sample forewent physician visits due to the patients’ inability to pay and about 6% of the sample reported foregoing hospital services. Thus, irrespective of the coping strategy (borrowing money or foregoing services) used by patients to deal with their inability to pay, the results showed that population groups with insufficient household resources (low income) and in frequent need of health care (poor health and chronic conditions) are the most vulnerable groups. The existence of an adequate exemption mechanism can only partly solve the problem of the inability to pay of vulnerable groups. This is because of the existence of informal payments in Bulgaria.

## Informal Payments for Health Care Services Continue to Exist in Bulgaria

Informal payments (both cash and in-kind informal payments) for health care services have a long tradition in Bulgaria (11–13). After the implementation of social health insurance, informal payments continued to exist despite the formal co-payments for services under the insurance scheme. Before the political changes in 1989, almost all informal payments were gifts in-kind. Informal cash payments emerged during the transition period and became rather widespread in the period before the social health insurance reform. The recent study conducted in Bulgaria in 2010 and 2011 (7) indicates the experience with informal payments after 10 years of official co-payments. The results show that in 2010, around 13% of the respondents who used outpatient services during the last 12 months, made informal payments (on average 45 EUR per year), and in 2011, around 10% of users report informal payments (on average 23 EUR per year). In 2010, approximately one-third of the respondents who were hospitalized during the last 12 months paid informally (on average 85 EUR per year), and in 2011, 18% of users made such payments (on average 108 EUR per year). Another study conducted in Bulgaria in 2010 (14) also confirms that about 10% of respondents have paid physicians informally in cash. Thus, the incidence of about 10–12% of the patients making informal payments has remained relatively constant during the years.

## Tolerance toward in-Kind Gifts for Health Care Services

Some studies conducted in Bulgaria before and after the reform analyze the attitudes and perceptions toward informal payments. The findings suggest the tolerance and acceptance of in-kind gifts. There are different expert opinions regarding the factors influencing the decision of patients to pay informally (15, 16). Taking into account, the three models proposed in the literature (17, 18), which explain the causes of informal payments by the joint effects of cultural perceptions, quality of governance, and the economic situation, we have put the emphasis on the cultural model to explain the high level of tolerance toward in-kind gifts. This model considers informal payments as a particular type of behavior of care seekers who express their gratitude in the form of gifts. The value of gifts is negligible and depends on the wealth of the patient. Thankful patient give in-kind gifts without any request or hint by the medical staff and in order to be truly a gift, it should be given after the service and not before. Therefore, a true expression of gratitude does not put a sizeable burden on patients and a necessary condition should be also that it is a voluntary act. Thus, the cultural model may be used to explain the positive attitude toward gifts in-kind observed in Bulgaria.

In contrast to in-kind gifts, informal payments in cash can have serious negative consequences. They may hinder access, create problems of the affordability to pay, may result in a refusal of treatment in the absence of payments, and lead to unnecessary medical interventions (19). The attention of policy-makers should be directed to the implementation of effective measures for their elimination, as well as the elimination of sizable in-kind gifts even though initiated by patients. In contrast to informal cash payments and sizable in-kind gifts, a true gratitude payment does not violate patient’s rights and dignity. The freedom of patients to give small in-kind gifts does not make them vulnerable. Therefore, this type of payment is difficult to eliminate. However, there should be clear regulations on what a thankful patient can give to medical personnel after the service provision (20).

## Conclusion

The parallel existence of formal and informal payments in Bulgaria has led to high out-of-pocket payments. Hence, there is a need to eliminate the existence of informal patient payments, which induce additional payment obligations for health care users. These payments are used by patients and health care providers as a means to overcome the poor service quality, to compensate the low remuneration of health care personnel, and to receive proper attention. However, the fight against these payments requires a set of different measures, persistency and strict control, and regular government investments in the improvement of service quality.

## Conflict of Interest Statement

The authors declare that the research was conducted in the absence of any commercial or financial relationships that could be construed as a potential conflict of interest.
